# Deciphering the Nutraceutical Potential of *Raphanus sativus*—A Comprehensive Overview

**DOI:** 10.3390/nu11020402

**Published:** 2019-02-14

**Authors:** Abinaya Manivannan, Jin-Hee Kim, Do-Sun Kim, Eun-Su Lee, Hye-Eun Lee

**Affiliations:** Vegetable Research Division, National Institute of Horticultural and Herbal Science, Rural Development Administration, Jeonju 55365, Korea; abinayamanivannan@gmail.com (A.M.); sayzinni@korea.kr (J.-H.K.); greenever@korea.kr (D.-S.K.); lus4434@korea.kr (E.-S.L.)

**Keywords:** Anticancer, Anti-diabetics, Antioxidants, Glucosinolates, Hepatoprotection

## Abstract

*Raphanus sativus* (Radish) belongs to the Brassicaceae family and is a widely consumed root vegetable all around the world. The nutritional and medicinal values of radishes have been proven by several researches. Extracts prepared from the aerial and underground parts of radishes have been used in the treatment of stomach disorders, urinary infections, hepatic inflammation, cardiac disorders and ulcers in folk medicine since the ancient times. The pharmaceutical potential of radishes is attributed to the presence of its beneficial secondary metabolites, such as glucosinolates, polyphenols and isothiocyanates. The present review has focused on the impact of radish extract administration under pathological complications, such as cancer, diabetes, hepatic inflammation and oxidative stress. In addition, a comprehensive view of molecular mechanism behind the regulation of molecular drug targets associated with different types of cancers and diabetes by the bioactive compounds present in the radish extracts have been discussed in detail.

## 1. Introduction

Diets enriched with plants have numerous health benefits to humans. It reduces the risk of various ailments, such as cancer, cardiovascular diseases, neurodegenerative disorders and aging related problems. Moreover, the plant-based diets supply plenty of antioxidants that are necessary for combating the harmful effects of free radicals, which are the inevitable byproducts of vital metabolisms. Plants consist of diverse pharmacologically important secondary metabolites. In contrast to primary metabolites, secondary metabolites occur in lower abundance and distribution and are deposited in specialized cells and organelles [1]. The plant based secondary metabolites can be classified into three major families, such as phenols, steroids and terpenes, and alkaloids [2]. Among the families, phenols, which are a wide range of compounds with one or more hydroxylated aromatic rings biosynthesized via shikimate pathway, are a major class of plant metabolites [3]. Generally, secondary metabolites aid the plant fitness by enhancing the plant–environment interaction. Consequently, the secondary metabolites in most cases acts as an antimicrobial and antioxidant in addition to being involved in plant defense against biotic and abiotic stresses.

Vegetables belonging to cruciferous plants have generated a wide range of dietary interest due to their higher nutritional and pharmaceutical potentials. Several reports illustrated that cruciferous vegetables consists of glucosinolates, phenolic compounds, tocopherols, carotenoids and ascorbic acid [4,5,6]. The principal antioxidative effects of the phytochemicals are manifested by the capability of the compound to scavenge the toxic free radicals or by hindering the oxidation of low-density lipoproteins [7,8,9]. Moreover, polyphenolic compounds have become the focus of present pharmaceutical industries, which is largely due to their health-promoting effects [5,6]. The radish (*Raphanus sativus* L., 2n = 18) is a well-known root vegetable crop belonging to the Brassicaceae family. The tap root of radishes has been consumed worldwide in the form of pickles, salads and curries due to its high nutritional values [6,7,8,10]. Apart from the roots, leaves and sprouts have also been reported to have nutritional and medicinal importance [9]. The extracts of radishes have been employed to treat stomach disorders, constipation, urinary infections, hepatic inflammation, cardiac disorders and ulcers in folk medicine since the ancient times [8]. In addition, various reports have recorded the antimicrobial [11,12], anticancer [13], antioxidant [14,15] and anxiety reducing properties [16] of radishes. The secondary metabolites with pharmaceutical benefits in radishes include glucosinolates, isothiocyanates and polyphenols [17,18,19]. Glucosinolates (GSL) are secondary metabolites that are exclusively found in cruciferous vegetables [4]. The chemical conformation of GSL possess ß-D-thioglucosides residue bonded to (Z)-N-hydroximinosulfate ester. GSLs are majorly classified into three types based on their precursor amino acids, such as aliphatic glucosinolates (AGSLs), aromatic glucosinolates (ArGSLs) and indolic glucosinolates (IGSLs) [20,21,22]. Recently, the GSLs have gained enormous interest in the pharmaceutical industry, especially in the designing of anticancer and antiinflammatory drugs. Hence, the present review will provide a comprehensive overview of the current research progress on the antioxidant, chemopreventive, hepatoprotective and antidiabetic properties of radishes. 

## 2. Antioxidant Effects of Radishes

The roots and leaves of radishes consist of vital nutritional values and diverse secondary metabolites with antioxidant properties. When compared with roots, leaves possessed higher levels of proteins, calcium and ascorbic acid whereas the total phenol contents were two-fold higher in leaves than roots which corresponded with the free radical scavenging ability [8]. The study has reported different forms of polyphenol constituents in the tissues. For instance, the elevated ranges of pyrogallol (free form) and vannilic acid (bounded form) were identified in roots whereas leaves consisted of epicatechin (free form) and coumaric acid in bounded form [8]. Interestingly, the leaves encompassed levels of flavonoids that were four-fold higher than in roots. Flavonoids are the major members of polyphenols with multiple hydroxyl groups and high free radical scavenging potential [23]. Hence, the leafy part of radishes can be considered as an excellent source of bioactive compounds with antioxidant potentials. A series of in vitro assays conducted by Wang et al. [24] illustrated the antioxidant and prooxidant properties of red radishes. The red radish has higher levels of anthocyanin dominated by the acylated pelargonidin derivative. In detail, the acylated pelargonidin derivatives present in the radish extract scavenged the 2,2′-azino-bis(3-ethylbenzothiazoline-6-sulphonic acid) (ABTS^+^) radicals and exerted free radical scavenging activity in a concentration-dependent manner. The ABTS^+^ assay is a predominant test to measure the antioxidant capacity of a compound using a spectrophotometer. Likewise, the acylated pelargonidin derivatives in radish had a higher reducing power potential. The ferric ion based reducing assay validated the free radical detoxification capacity of radishes. In general, the metal chelation hinders the formation of ROS and the compounds that possess the ability to chelate iron are considered to combat the ROS. Apart from the above assays, the prooxidant capacity of acylated pelargonidin derivatives in the radish extracts was determined using the plasmid DNA damage assay. The roles of radish extract as the antioxidant or prooxidant is determined by the concentration and the reaction condition. In addition, the prooxidant activity of radish extract is also based on the nature of the radicals and the prooxidant activity of radish varied between the cancer cells and plasmid DNA. However, deeper insights on the molecular mechanisms that trigger the prooxidant and antioxidant capacity need to be obtained by future researches. Anthocyanins are well-known antioxidants involved in the donation of hydrogen, metal chelation and protein binding [25,26,27]. In addition, anthocyanin also act as a chemoprotective agent by triggering the phase II antioxidant enzymes, preventing cell proliferation and enhancement of apoptosis [28,29,30,31,32]. Secondary metabolites with antioxidant properties identified in radishes have been listed in Table 1.

## 3. Hepatoprotective Effects of Radishes

The hepatoprotective effects of the radish extract have been recorded by various researchers [36,42,43,44,45,46,47]. Bioactive compounds, such as indole-3-carbinol, 3-[ethoxy- (methylthio)methyl]-2-pyrrolidinethione and 3-(E)-(methylthio)-methylene-2-pyrrolidinethione, present in the radish root and sprouts decreased the severity of fatty liver disease in mouse models [48]. Moreover, the extracts of black radishes alleviated the negative effects of carbon tetrachloride (CCl_4_)-induced liver injury in rats [43]. The administration of a radish extract resulted in the inhibition of lipid accumulation caused by the oxidative stress. According to Ahn et al. [43], the radish extract upregulated the expression of cytochrome P 450 (CYP) CYP2E1, nuclear factor erythroid 2-related factor-2 (Nrf-2) and Heme oxygenase-1(HO-1) in a concentration-dependent manner. Moreover, the report suggested the possible mechanism behind the hepatoprotective effects rendered by radish could be due to the mediation of Nrf-2/HO-1 antioxidant pathway [43]. In general, Nrf-2 targets the HO-1 molecule, which plays a vital role in the amelioration of oxidative stress and helps in the regulation of genes associated with inflammation, cytoprotection and antioxidant activity [49]. Similarly, the ingestion of fresh radish juice reduced the hepatotoxicity induced by CCl_4_ in albino rats by the prevention of lipid peroxidation, which replenished the levels of non-protein sulfhydryl moiety (NP-SH) and enhanced the detoxification system of liver [50]. In addition, the phytochemical characterization of the fresh radish juice revealed the presence of hepatoprotective sulphurated compounds, phenols and terpenoids [50]. Similarly, Lee et al. [47] evidenced the hepatoprotective effects of radish enzyme extract in human liver derived cells (HepG2) and rats against tarcine and CCl_4_ induced hepatotoxicity. The histopathological investigations and biochemical analysis revealed that the radish enzyme extract efficiently provided protection against membrane fragility and reduced the leakage of glutamate oxaloacetate (GOT) and glutamate pyruvate transaminase (GPT) [47]. In liver, the disruption of cellular integrity by CCl_4_ increases the activities of GOT and GPT enzymes, which are considered to be biomarkers for the identification of liver damage [51,52]. Likewise, the triglycerides (TG) and total cholesterol (TC) in serum can indicate the status of the liver damage [53,54]. The supplementation of radish enzyme extract significantly reduced the levels if TC and TG in CCl_4_-treated rats [47]. Thus, the decrease in the activities of GOT and GPT and the levels of TC and TG by radish enzyme extract suggested the hepatoprotective potential of radishes.

## 4. Anticancer Effects of *R. sativus*

In recent days, the interest in diets enriched with potential bioactive natural compounds with anticancer properties is increasing. Ingestion of cruciferous vegetables has significant benefits of chemoprevention due to the presence of secondary metabolites, such as glucosinolates, which are highly noted for their anticancer properties. Several studies have recorded the antiproliferative effects of isothiocyanates, the hydrolyzed forms of glucosinolates in different forms of cancers [12,13,41,55]. According to Rampal et al. [56], the isothiocyanates exhibit multiple anticancer mechanisms with pharmaceutical interest, such as regulation of detoxification enzymes, activation of apoptosis and prevention of cell cycle progression. In this section, a comprehensive overview on the effects of radish extracts on various cancers was provided. 

### 4.1. Liver Cancer

The extract of Spanish black radishes significantly inhibited the proliferation of HepG2 cells by the regulation of phase I and phase II detoxification system [41]. The anticancer property of the extract has been attributed to the glucosinolate compounds, which are namely glucoraphasatin and 4-methylthio-3-butenyl isothiocyanate. According to the report, the crude extract improved the activity of phase II detoxification enzymes, such as quinone reductase, heme oxygenase 1 and thioredoxin reductase 1. In addition, the mRNA levels of phase I detoxification enzymes, such as cytochrome P450 (CYP) 1A1, CYP1A2 and CYP1B1, were also increased upon the addition of radish extracts. The results suggested that the radish extract activated the detoxifying enzymes by activating the aryl hydrocarbon receptor (AhR) and NF-E2-related factor 2 (Nrf2) pathways [57,58,59,60,61,62]. However, the activation of phase I detoxification appeared to be of concern because of the reactive intermediates formation, which increases the toxicity, but the synergistic induction of phase II detoxification system is necessary for the elimination of toxic compounds [41]. 

### 4.2. Colon Cancer

The extracts of Thai rat tailed radishes displayed effective cytotoxicity against colon cancer cell line (HCT116) [13]. The occurrences of bioactive phytochemicals, such as sulforaphane and sulforaphane, have been identified in the extract using gas chromatography–mass spectrometry (GC–MS). The radish extract induced apoptosis in the colon cancer cell line. Sulforaphane induced free radicals in cancer cells and was involved in the disruption of microtubule polymerization [63,64,65]. In general, the cancer cells has higher levels of basal ROS. Thus, tailoring the ROS to target the cancer cells could be a potential strategy for designing anticancer drugs with higher selectivity. The administration of sulforaphane and sulforaphene resulted in the death of cancerous cells whereas the normal cells were unaffected. The presence of an active antioxidant mechanism in normal cells could have prevented the death of normal cells. In addition, the sulforaphane triggered the intrinsic and extrinsic apoptosis pathway in cancer cells. For instance, in the intrinsic pathway, sulforaphane induced the regulation of mitochondrial membrane proteins and enhanced the proapoptotic protein expression whereas it decreased the levels of antiapoptic proteins, resulting in the activation of caspase cascade [64]. Similarly, the administration of sulforaphane improved the apoptosis by the induction of TNF-related apoptosis inducing ligand (TRAIL) and alleviation of extracellular signal-regulated kinase (ERK) and Akt in the extrinsic pathway [63,66,67]. On the other hand, the antimutagenic effect of sulforaphene is robust compared to sulforaphane [68]. According to Papi et al. [69], the sulforaphene elicited the intrinsic apoptosis pathway in human colon cancer cell lines although the exact molecular rationale studies behind the anticancer activity of sulforaphene is still in rudimentary stages. Both sulforaphane and sulforaphene contains highly electrophilic central atoms, which interact with nucleophilic cellular targets, such as glutathione synthase hydrogenase (GSH) and cysteine amino acid in Keap1 protein involved in the stabilization of Nrf2 [69]. Apart from the induction of cytotoxicity and apoptosis, the bioactive compounds stimulated the phase II enzymes, which aids in the detoxification of carcinogens.

### 4.3. Breast Cancer

The aerial extract of radishes actively induced cytotoxicity in the breast cancer cell line (MDA-MB-231) via the ErbB-Akt pathway [70]. The epidermal growth factor receptor (EGFR) is a potential oncogene in breast cancer, with the repression of EGFR amplification reported to have antiproliferative benefits in breast cancer [71]. EGFR is composed of ErbB1, ErbB2, ErbB3 and ErbB4 proteins, an increase in the overexpression of ErbB proteins has been linked to the development of breast cancer [72]. In general, the docking of EGFR ligands to the EGF receptors initiates the formation of heterodimers, which triggers the autophosphorylation of tyrosine kinase. The active phosphorylated EGFRs serves as the binding sites for the proteins involved in signaling cascades for cellular proliferation and survival [73,74]. The application of radish aerial extracts downregulated the mRNA and protein expression levels of ErbB2 and ErbB3 in MDA-MB-231 cell lines [70]. Moreover, the EGF receptor-ligand interaction activates the PI3K/Akt pathway, which plays an important role in tumorigenesis [75,76,77]. Akt is involved in the enhancement of cell survival and suppression of cell death [78]. In addition, Akt is also involved in the phosphorylation of caspase 9, Bad and proapoptotic transcription factors, which prevents the antiapoptotic property [79,80,81]. The administration of a radish extract also decreased the expression of Akt in a dose-dependent manner, thus increasing the antitumor activity [70]. Hence, the aerial extract of radishes was proved to be a valuable source for antitumor drug discovery. Likewise, the active isothiocyante component sulforaphene in radish significantly reduced the viability of SKBR-3 breast cancer cell line with less toxicity towards normal cells [82]. According to Pawlik et al. [82], sulforaphene arrested the cell cycle in G2/M phase, disrupted the organization of cytoskeleton, decreased the colonization of cancer cells and induced apoptosis. The cells cultured with sulforaphene displayed increased Bax protein (proapoptotic protein) whereas the Bcl-2 (antiapoptotic) levels were decreased. Similarly, the level of ADRP proteins, which is involved in the lipid coating during cellular stress due to mitochondrial dysfunction, increased upon sulforaphene treatment [55,83]. Previous reports suggested that the sulforaphene treatment results in the disintegration of mitochondrial potential and leads to the inhibition of mitochondrial respiratory chain complexes I and III [84,85]. In addition, sulforaphene also reduced the caspase-dependent degradation of PARP protein, which can be related to the existence of other suicidal proteases [86,87]. Overall, the sulforaphene present in the radish extract can be a potential anticancer agent with higher efficacy to cancer cells and has comparatively less toxicity to normal cells.

In addition, the radish extract prevented the deleterious effects of zearalenone (zen), a mycotoxin synthesized by *Fusarium* spp [88]. The zen toxicity has been widely associated with the liver and breast cancer. The administration of radish extract significantly improved the immune cells, such as lymphocytes, immunoglobulins, T-cells, B-cells and interleukins, which were able to act against zen toxicity. The oral ingestion of radish extracts enhanced the release of tumor necrosis factor (TNF-α), which is a vital antitumor drug candidate under study. Overall, the intake of radish extract detoxified the zen toxicity by the improvement of immune cells, inhibition of lipid peroxidation and elicitation of TNF-α.

### 4.4. Cervical, Lung and Prostate Cancer

**Figure 1:**
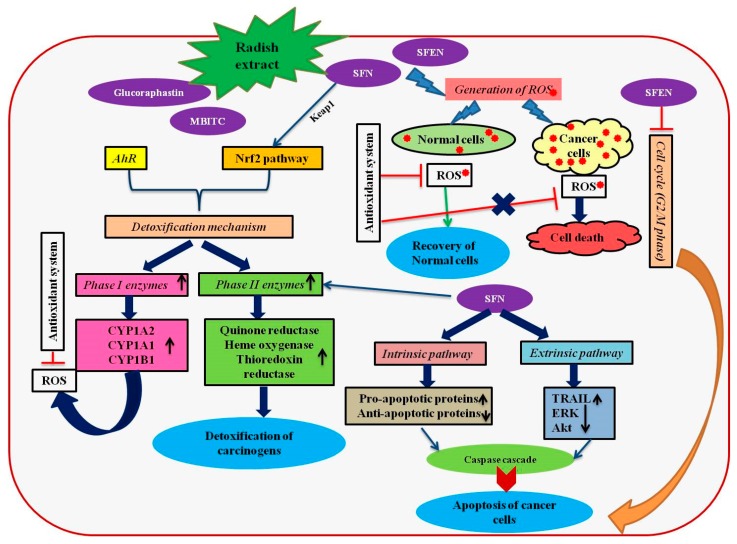
A schematic representation of anticancer mechanisms induced by the bioactive compounds present in radish extracts. The figure has been conceived based on the interpretation of the literatures cited in Section 4. AhR, aryl hydrocarbon receptor; Akt, alpha serine-threonine protein kinase; CYP, cytochrome P450; ERK, extracellular signal-regulated kinase; MTBITC, 4-(methylthio)-3-butenyl isothiocyanate; Nrf2, NF-E2-related factor 2; ROS, reactive oxygen species SFN, sulforaphane; SFEN, sulforaphene; TRAIL, TNF-related apoptosis inducing ligand.

The chemopreventive effects of different parts of radishes have been evaluated in cervical (HeLa), lung (A549) and prostate (PC-3) and breast (MCF-7) cancer cell lines by Beevi et al. [89]. The report illustrated the molecular rationale behind the radish extract mediated apoptosis in cancer cell lines. In detail, the hexane extract obtained from the roots of radishes was comprised of isothiocyanates (ITCs), such as 4-(methylthio)-3- butenyl isothiocyanate (MTBITC), 4- methylthio)-3-butyl isothiocyanate (erucin), 4-methylpentyl isothiocyanate, 4-pentenyl isothiocyanate and sulforaphene. Radish extracts caused apoptosis in both p53 deficient and proficient cell lines, which denoted the effect of extract induced apoptosis signaling irrespective of the status of p53 in the cells. Additionally, the apoptosis process involved the interaction of Bcl2family genes and caspase-3 activation [89]. Interestingly, the radish extract selectively targeted the cancer cells without affecting the normal cells, which is a prerequisite for the potential anticancer drug. The radish extract treatment resulted in the detachment of cancer cells, inhibition of cell elongation, induction of cell shrinkage and DNA fragmentation [89]. Variation in the expression levels of genes involved in apoptosis have been determined in cancer cell lines with different tissue lineages treated with radish extract [55,89]. Similarly, the in vivo administration of sulphoraphene actively inhibited tumor growth in mice [90]. The ingestion of sulphoraphene in Balb/C mice with lung cancer prevented the tumor growth by the inhibition of P13K-AKT signaling, reducing the expression of PTEN and ceasing the phosphorylation of AKT in mice. Thus, the study evidenced the anticancer effects of a major isothiocyanate present in radishes in the animal model. Different mechanisms exhibited by radish extracts to prevent cancer cell proliferation have been illustrated in Figure 1. 

## 5. Antidiabetic Effects of Radish

**Figure 2:**
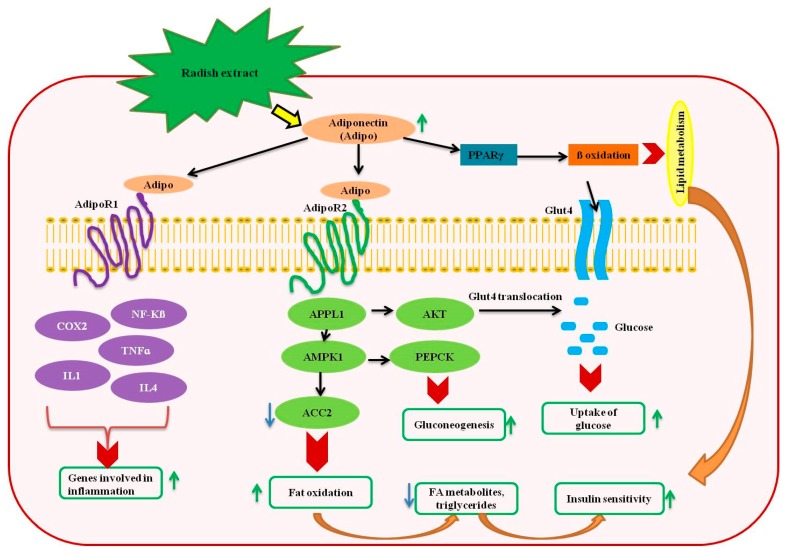
A schematic representation of anti-diabetic mechanisms induced by the radish extracts. The figure has been conceived based on the interpretation of the literatures cited in Section 5. ACC2, acetyl-CoA carboxylase 2; APPL1: adaptor protein, phosphotyrosine interaction, pH domain and leucine zipper containing 1; ADIPOR, adiponectin receptors; Akt, alpha serine-threonine protein kinase; COX2, cyclooxygenase 2; AMPK, adenosine monophosphate-activated protein kinase; NF-kβ, nuclear factor kappa-light-chain-enhancer of activated B cells; TNFα, tumor necrosis factor alpha; IL, interleukin; PPARγ, peroxisome proliferator-activated receptor gamma; PEPCK, phosphoenolpyruvate carboxykinase.

Recently, the incidence of diabetes has become a major health threat worldwide. Diabetes is one of the leading causes of mortality in humans [91]. In order to prevent the disease, several countries have adapted various therapeutic approaches and scientists are continuously striving to discover potential antidiabetic agents [92]. However, the need to circumvent the uncontrolled homeostasis of glucose metabolism has led to the search of plant-based antidiabetic compounds [92,93,94]. The usage of radish extracts in the treatments of digestive or stomach-related ailments since the ancient times has given a clue for the occurrence of phytochemicals with antidiabetic properties in radishes. The water soluble extract of radish displayed hypoglycemic properties due to the presence of insulin-like polyphenols or glucose-inhibiting compounds [95,96]. Likewise, several studies have recorded the antidiabetic effects of radish in the in vitro and in vivo environment [15,97,98]. The antidiabetic nature of radish extracts can be due to the following mechanisms: (a) regulation of glucose related hormones, (b) prevention of diabetes-induce oxidative stress and (c) balancing the glucose uptake and absorption [92]. The radish extracts enhanced the synthesis of adiponectin, a central regulatory protein involved in the regulation of lipid and glucose metabolism secreted by adipose tissue [99,100]. Adiponectin increases insulin sensitivity and enhances the bodyweight reduction [101]. It synchronizes various metabolic processes and aids in the maintenance of glucose uptake and lipid oxidation processes [101,102]. Moreover, adiponectin regulates several genes involved in inflammation, cellular proliferation, cell death, endosomal trafficking and chromatin remodeling [103]. An increase in the production of adiponectin triggers the adiponectin receptors (ADIPOR1 and 2) and peroxisome proliferator-activated receptor gamma (PPARγ) [101]. The ADIPOR1 stimulates the genes involved in inflammation and regulation of oxidative stress whereas ADIPOR2 activates adaptor protein, phosphotyrosine interaction, pH domain and leucine zipper containing 1 (APPL1), which in turn increases the expression of genes that are vital for gluconeogenesis and glucose uptake [104,105]. On the other hand, PPARγ maintains the beta oxidation in lipid metabolism. The adiponectin interaction with its receptors results in the phosphorylation of acetyl-CoA carboxylase 2 (ACC2), which increases the oxidation of fatty acids and enhances the insulin sensitivity [101,106,107]. In addition, the increase in the ROS levels have been alleviated by adiponectin mediated regulation of transcription of genes involved in antioxidant machinery, such as superoxide dismutase (SOD) [108]. Overall, the stimulatory effects of radish extract on adiponectin could be an important tactic to combat the diabetes. A detailed illustration of the possible mechanisms involved in the adiponectin mediated prevention of diabetes by radish extract has been shown in Figure 2.

Similarly, the extracts of Japanese radish sprouts displayed antidiabetic activity in streptozotocin-induced diabetic rats [97]. In addition, the administration of radishes decreased the starch-stimulated glycemic load, which provided evidence for the prevention of diabetes [109]. Moreover, the occurrence of polyphenols, such as catechin in radishes, improved the insulin secretion [110]. Apart from the regulation of glucose metabolism, the antioxidant activity of radish prevented the oxidative stress induced by diabetes. For instance, radishes enhanced the synthesis of superoxide dismutase (SOD) like proteins and endogenous glutathione and catalase enzymes to scavenge the free radicals and prevented the peroxidation of lipids under diabetic conditions [111,112]. Similarly, radish extracts with pelargonidin (anthocyanin derivative) aid in the antioxidant activity by decreasing the generation of free radicals and formation of a thiobarbituric acid reactive moiety [113]. Another important antioxidant compound, sulforaphane (isothiocyanate), induces the phase II antioxidant enzymes and maintains the redox balance upon oxidative stress [114]. In addition, the nutritional and secondary metabolites content of radishes can be varied by using a different method of processing for consumption [115]. Overall, the radish extracts consist of high antidiabetic values although the exact mechanism associated with antidiabetic properties has to be determined in the future, which can be utilized in the antidiabetic drug designing pipelines. 

## 6. Conclusions

Radishes is of great pharmaceutical importance, most of which has been attributed to its antioxidant property. The administration of radish extracts under numerous pathological conditions aids in the recovery of diseases and in the prevention of harmful ailments because of their attributed bioactivities. Bioactive compounds present in different parts of radishes, such as leaves, sprouts, stem and roots, act on a variety of potential drug targets associated with ailments, such as cancer, inflammation, liver injury and diabetes. However, the in-depth molecular mechanistic studies are required to address the regulatory roles of bioactive compounds in radish extracts. In future, researches focusing on the determination and pharmacokinetic elucidation of the bioactive compounds in radishes could facilitate the designing of plant based drugs for life threatening disorders, such as cancer and diabetes. Overall, the knowledge gained from the present researches in radish should be utilized in the discovery of novel drug molecules with higher efficacy towards drug targeting with less side effects.

## Figures and Tables

**Table 1:** List of secondary metabolites with antioxidant properties identified in different parts of radish.

Name	Class	Tissue	Reference
1,2-dihydroxyferuloyl-gentiobiose	Phenolic acid	Leaves	[33]
13Z-ß-Carotene	Carotenoids	Sprouts	[34]
3-Butenyl isothiocyanate	Isothiocyanates	Pod & flower	[13]
4-methoxyglucobrassicin	Glucosinolate	Sprouts	[35]
4-OH-glucobrassicin	Glucosinolate	Sprouts	[35]
6-Prenyl-naringenin	Flavonone	Root	[33]
6,7,30,40-Tetrahydroxyisoflavone	Isoflavonoids	Leaves	[33]
9Z-ß–carotene	Carotenoids	Sprouts	[34]
α-Carotene	Carotenoids	Sprouts	[34]
Antheraxanthin	Carotenoids	Sprouts	[34]
Anthocyanin-3-O-(cinnamoyl) sophoroside-5-O-glucoside derivatives	Anthocyanin	Sprouts	[36]
Anthocyanin 3-O-sophoroside-5-O-(malonyl) glucoside derivatives	Anthocyanin	Sprouts	[36]
Apigenin	Flavonoids	Sprouts & seeds	[37]
Apigenin-7-O-neohesperidoside	Flavone	Leaves	[33]
Apigenin-7-O-rutinoside	Flavone	Leaves	[33]
Caffeic acid	Phenolic acid	Sprouts & seeds	[37]
Caffeoylmalic acid	Polyphenols	Leaves	[38]
Chrysoeriol-7-O-apiosyl-glucoside	Flavone	Leaves	[33]
Cyanidin-3-O-caffeoyl-p-coumaroyl-sophoroside- 5-O-glucoside	Anthocyanin	Root	[33]
Cyanidin-3-O-di-p-coumaroyl-sophoroside-5-Omalonylglucoside	Anthocyanin	Root	[33]
Cyanidin-3-O-glucoside	Anthocyanin	Leaves	[33]
Cyanidin-3-O-p-coumaroyl-feruloyl-sophoroside- 5-O-glucoside	Anthocyanin	Root	[33]
Cyanidin-3-O-rhamnoside	Anthocyanin	Leaves	[33]
Cyanidin-3-O-sophoroside-5-O-glucoside	Anthocyanin	Leaves	[33]
Cyanidin-3-O-sophoroside-5-O-malonylglucoside	Anthocyanin	Leaves, root	[33]
Cyanidin-3-O-xylosyl-p-coumaroyl-glucosylgalactoside	Anthocyanin	Leaves	[33]
Delphinidin-3-O-rutinoside	Anthocyanin	Root	[33]
Dihydro-caffeoyl-3-O-glucuronide	Phenolic acid	Root	[33]
Dihydro-kaempherol-3-O-rutinoside	Dihydroflavonol	Leaves	[33]
E- ß –carotene	Carotenoids	Sprouts	[34]
Ferulic acid	Phenolic acid	Aerial parts	[39]
Ferulic acid	Phenolic acid	Sprouts & seeds	[37]
Feruloylmalic acid	Phenolic acid	Leaves	[38]
Gallic acid	Phenolic acid	Sprouts & seeds	[37]
Genistin	Isoflavonoids	Leaves	[33]
Glucobrassicin	Glucosinolate	Sprouts	[40]
Glucodehydroerucin	Glucosinolate	Sprouts	[40]
Glucoraphasatin	Glucosinolate	Whole plant, sprouts	[40,41]
Glucoraphenin	Glucosinolate	Sprouts	[42]
Indole-3-carbinol	Isothiocyanates	Sprouts	[15]
Isorhamnetin-3-O-p-coumaroyl-caffeoylsophorotrioside- 7-O-malonyl-glucoside	Flavanol	Root	[33]
Isorhamnetin-3-O-p-coumaroyl-sophorotrioside- 7-O-glucoside	Flavanol	Leaves	[33]
Kaemferol	Flavonoids	Sprouts & seeds	[37]
Kaempferitrin	Polyphenols	Leaves	[38]
Kaempferol-3-O-caffeoyl-sophoroside-7-Oglucoside	Flavanol	Root	[33]
Kaempferol-3-O-feruloyl-sophoroside-7-Oglucoside	Flavanol	Root	[33]
Kaempferol-3-O-glucoside	Flavanol	Leaves	[33]
Kaempferol-3-O-glucosyl-rhamnosyl-glucoside	Flavanol	Leaves	[33]
Kaempferol-3-O-p-coumaroyl-sinapoylsophorotrioside-7-O-malonyl-glucoside	Flavanol	Leaves, root	[33]
Kaempferol-3-O-p-coumaroyl-sophorotrioside- 7-O-glucoside	Flavanol	Leaves	[33]
Kaempferol-3-O-rhamnoside(I)	Flavanol	Leaves	[33]
Kaempferol-3-O-rutinoside	Flavanol	Leaves	[33]
Kaempferol-3-O-xylosyl-rutinoside	Flavanol	Leaves	[33]
Lutein	Carotenoids	Sprouts	[33]
Luteolin-7-O-glucoside	Flavone	Leaves	[33]
m-Coumaric acid	Phenolic acid	Leaves	[33]
Methylgalangin	Flavanol	Leaves	[33]
Naringenin-40-O-glucuronide	Flavonone	Leaves	[33]
Naringenin-7-O-glucuronide	Flavonone	Leaves	[33]
p-Coumaric acid	Phenolic acid	Sprouts & seeds	[37]
p-Coumarylmalic acid	Polyphenols	Leaves	[38]
Pelargonidin-3-O-caffeoyl-caffeoyl-diglucoside-5-O-malonyl-glucoside	Anthocyanin	Root	[33]
Pelargonidin-3-O-feruloyl-diglucoside-5-Oglucoside	Anthocyanin	Leaves	[33]
Pelargonidin-3-O-p-coumaroyl-diglucoside-5-Oglucoside	Anthocyanin	Leaves	[33]
Pelargonidin-3-O-sambubioside	Anthocyanin	Leaves	[33]
Protocatechuic acid	Phenolic acid	Sprouts & seeds	[37]
Quercetin	Flavonoids	Sprouts & seeds	[37]
Quercetin-3-O-p-coumaroyl-sophoroside-7-Oglucoside	Flavanol	Leaves	[33]
Quercetin-3-O-rhamnoside	Flavanol	Leaves	[33]
Quercetin-3-O-rhamnosyl-galactoside	Flavanol	Leaves	[33]
Sinapic acid	Phenolic acid	Sprouts & seeds	[37]
Spinacetin-3-O-(200-p-coumaroyl-glucosyl)(1-6)-(apiosyl(1_2))-glucoside	Flavanol	Root	[33]
ß-Cryptoxanthin	Carotenoids	Sprouts	[34]
Stigmasterol	Phytosterol	Aerial parts	[39]
Sulforaphane	Isothiocyanates	Pod & flower	[13]
Sulforaphene	Isothiocyanates	Pod & flower	[13]
Violaxanthin	Carotenoids	Sprouts	[34]
Zeaxanthin	Carotenoids	Sprouts	[34]
β-sitosterol	Phytosterol	Aerial parts	[39]
β-sitosterol-3-β-O-D-glucopyranoside	Phytosterol	Aerial parts	[39]
